# Impact of Future Work Self on Employee Workplace Wellbeing: A Self-Determination Perspective

**DOI:** 10.3389/fpsyg.2021.656874

**Published:** 2021-07-15

**Authors:** Zhongqiu Li, Yang Yang, Xue Zhang, Zhuo Lyu

**Affiliations:** ^1^College of Economics and Management, Northeast Agricultural University, Harbin, China; ^2^School of Management, Harbin Institute of Technology, Harbin, China; ^3^College of Philosophy, Law and Political Science, Shanghai Normal University, Shanghai, China

**Keywords:** future work self, self-management, person-organization fit, self-determination theory, employee workplace wellbeing

## Abstract

This study examines the association between future work self and employee workplace wellbeing by proposing a moderated mediation model. On the basis of the self-determination theory, self-management is identified as the mediator, and person–organization fit is recognized as the moderator in this study. We collected two waves of data from 239 Chinese employees. The results of the analysis revealed that the future work self is related to greater employee workplace wellbeing, and self-management mediates the links between them. We also found that the person–organization fit strengthens the positive relationship between future work self and self-management, and the indirect effect of future work self on employee workplace wellbeing through self-management. The results of this study extend the antecedents of employee workplace wellbeing and highlight the importance of future work self in current work-related output.

## Introduction

Humans tend to desire and pursue a better experience, such as wellbeing (Wiklund et al., 2019), especially in the new economic era with rising living standards. Employee workplace wellbeing is defined as employee cognition of satisfaction at work, and the emotional and psychological experience and health status expressed at workplaces (Zheng et al., 2015). Moreover, employee workplace wellbeing, which can be viewed as positive sentiments experienced at work, can be used as an indicator to measure the mental health of staff in an organization (Chari et al., 2018) and is very important for ensuring the success of an organization (Daniels and Harris, 2000; Su and Swanson, 2019). Studies have shown that employee workplace wellbeing can affect attitudes and behaviors (Sharma et al., 2016), such as supportive green behaviors (Su and Swanson, 2019), loyalty (Chiu et al., 2013), and turnover intention (Gong et al., 2018). Employee workplace wellbeing can also yield outcomes at the organizational level, such as improved organizational performance (Taris and Schreurs, 2009). Hence, in recent years, employee workplace wellbeing has become a key concern for both academics in the field of organizational behavior and managers in organizations (Zheng et al., 2015). Given its significance, the study on employee workplace wellbeing has continued to increase over the past few decades.

Relating to the prevalence of employee workplace wellbeing, researchers have primarily focused on its favorable antecedents, such as individual-, group-, leader-, and organization-level workplace resources (Nielsen et al., 2017). In particular, the importance of motivational resources in influencing work motivation and employee workplace wellbeing has been emphasized by organizational psychology scholars (Boncquet et al., 2020). The future work self is defined as an image or a reflection of an individual in future work expectations and ambitions (Strauss et al., 2012). It is the embodiment and extension of the individual “possible self” in the workplace (Markus and Nurius, 1986). Strauss et al. (2012) hold that it includes two dimensions, namely, salience and elaboration. An elaborate future work self is complicated and comprises manifold components, which is difficult to measure (Strauss et al., 2012). Therefore, considering the research theme of our investigation and the critical role of the salient future work self in the motivation process of individual self-concept, we focused on the salient dimension of future work self in this study (i.e., the degree of clarity and easy imagination of the future work self, contributing to positive feelings about the self; Strauss et al., 2012). Prior empirical studies have also used the salient dimension to measure future work self (Taber and Blankemeyer, 2015; Zhang et al., 2016; Guan et al., 2017; Yang et al., 2019). When individuals perceive their possible self, they experience increased personal motivation, which has an effect on their career-related behaviors (Hoyle and Sherrill, 2006). Earlier studies have demonstrated that the future work self positively predicts career-related outcomes (Taber and Blankemeyer, 2015), career adaptability (Guan et al., 2014), performance (Lin et al., 2016; Oh, 2020), the meaning of life, calling (Zhang et al., 2016), employee creativity (Yang et al., 2019), and job search behaviors (Kao et al., 2020).

Although many examples of research have suggested the benefits of employees having a future work self, most studies focus on its future-oriented outcomes (Guan et al., 2014; Kao et al., 2020). However, as Lin et al. (2016) noted, the future work self has current work-related outputs such as job performance (Lin et al., 2016) and creativity (Yang et al., 2019). This study echoes the current demand for the deeper analysis of specific dependent variables that can be influenced by future work self (Lin et al., 2016), especially current work-related outputs such as employee workplace wellbeing. The self-determination theory suggests that individuals tend to long for goals that sustain their need for satisfaction (Deci and Ryan, 2000). The need for competence, autonomy, and relevance predicts mental health, such as wellbeing (Deci and Ryan, 2000). Furthermore, the future work self focuses on future, positive, concrete work, prompting future-oriented cognition for realizing the idealized self (Strauss et al., 2012), thus influencing the attitudes and behaviors of individuals. Hence, this study emphasizes the impact of having a future work self, particularly on employee workplace wellbeing.

This study proposed the mediating role of self-management, reflecting the skills of people in self-observation that was developed and adapted to the organizational environment to improve the realization of the expected behaviors of employees (Frayne and Latham, 1987). The self-management of employees at the workplace refers to how employees can control their behavior without supervision (Breevaart et al., 2014), and it is a self-directed change technique that can enhance self-regulation through purposeful implementation (Renn et al., 2011). As the future work self represents the expectations of a person for work in the future, this may create a difference between the current self and the ideal self (Strauss et al., 2012). By assessing the gap between the present self and the ideal self, the identification of behavior–goal discrepancies is positively related to a subsequent increase in goal-directed efforts (Kernan and Lord, 1990). Individual positive work behaviors, such as experiencing increased self-management, can yield beneficial consequences for employee workplace wellbeing (Schueller and Seligman, 2010).

To acquire more profound insights into the relationship between future work self and self-management, we also sought to identify the key boundary conditions that influence any causal relationship. A crucial part of this effort was testing the moderating effect of the person–organization fit. The self-determination theory indicates that the context can affect employee cognition and behavior (Deci and Ryan, 2000; Deci et al., 2017). Individuals are more likely to form active behavioral motivations and produce positive behavioral outcomes in contexts that favor the fulfillment of the basic psychological needs of the individual. This study views a high person–organization fit as allowing employees to address their basic psychological needs. In essence, this study tests the moderating effect of the person–organization fit, i.e., whether the positive impact of future work self on self-management and thus employee workplace wellbeing will be stronger for employees with high person–organization fit.

**Figure 1:**
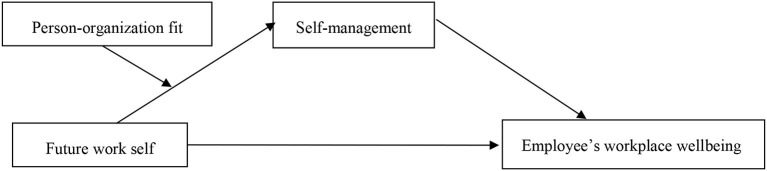
Research model.

The contributions of this study are 3-fold. First, using the self-determination theory, we confirmed that the future work self can affect employee workplace wellbeing. This finding supports recent calls to examine the motivational antecedents of wellbeing (Boncquet et al., 2020). Furthermore, the finding adds to extant studies and echoes a desire for more research on the relationships of future work self with current outcomes (Lin et al., 2016). Second, we advanced the literature by considering self-management as the mechanism expanding the understanding of why future work self affects employee workplace wellbeing. Third, we contributed to the literature by testing the moderating effect of the person–organization fit on the indirect impact of future work self on employee wellbeing through self-management. We identified the boundary condition of the benefits of future work self. The research model of this study is depicted in Figure 1.

## Theory and Hypotheses

### Future Work Self and Employee Workplace Wellbeing

Wellbeing is a comprehensive concept of happiness that originates from the field of positive psychology (Ryan and Deci, 2001) and includes many types (Zheng et al., 2015). Among them, employee workplace wellbeing, such as the psychological experience and satisfaction state of emotion on the work level, has gradually attracted the attention of scholars (Zheng et al., 2015).

We suggest that having a salient future work self can enhance employee workplace wellbeing. The first possible reason is that individuals may continue to set specific goals to achieve an ideal future. Individuals can be influenced by personal motivational factors (Miller and Brickman, 2004; Ryan and Deci, 2017). A future work self that envisions the positive self-working can be regarded as the motivational factors of individuals and may push them to pursue a positive identity at work (Strauss et al., 2012). For the sake of reducing the disparity between reality and their future work self, individuals will make specific efforts to establish certain goals. Individuals pursuing internal goals tend to show more positive results (Kasser and Ryan, 1996). The anticipation of attaining valued goals in the future, in turn, has been shown to have salutary effects on, first, mental health and, subsequently, employee workplace wellbeing (Prenda and Lachman, 2001; Kooij et al., 2018).

A second way whereby future work self may affect employee workplace wellbeing is by meeting the basic needs of individuals. The self-determination theory holds that individuals have three basic needs, namely, autonomy, competence, and relatedness. When these needs are met, people enter into an autonomous mode of self-regulation that fosters intrinsic engagement and employee workplace wellbeing (Deci and Ryan, 2000; Akan et al., 2020). The future work self may push individuals to have high expectations and a strong desire for work (Strauss et al., 2012). Therefore, they are intrinsically motivated, and their autonomy needs can be met (Deci and Ryan, 2008). Individuals with a salient future work self are eager to realize the value of life through work (Strauss et al., 2012) and actively face problems in their work. When they solve work problems to realize their future work selves, their competence needs can be satisfied (Deci and Ryan, 2000). When employees believe that their activities are valuable, their wellbeing will increase (Patrick et al., 2007). Earlier studies have shown that individuals who can anticipate and plan for future outcomes find it easier to experience wellbeing (Kooij et al., 2018). In addition, their future work self can improve employee workplace wellbeing by meeting their basic needs. We thus hypothesized the following:

Hypothesis 1: Future work self has a positive effect on employee workplace wellbeing.

### Mediating Role of Self-Management

As a self-directed change technique, self-management strengthens self-regulation by purposefully implementing and coordinating personal goal setting, monitoring, and operation (Renn et al., 2011). Personal goal setting refers to setting goals to enhance desired behaviors (Frayne and Geringer, 2000). Monitoring means that the behavior of the target is regularly monitored and compared with individual improvement goals (Renn et al., 2011). Finally, individuals operate in their roles according to the dual situation of self and environment (Frayne and Geringer, 2000; Renn et al., 2011).

On the basis of the self-determination theory, we argued that the future work self could strengthen self-management. First, the future work self helps employees seek the goals of work and even life, feel responsible for their work, and understand that work is the way to realize their value, enhancing employee self-management. The self-determination theory holds that the behavior of individuals can be affected by identified regulation, which is the belief of individuals that their behavior is essential to and is associated with self-worth and goals (Deci and Ryan, 2000). Individuals also rely on memories, present experiences, and aspirations to give them a sense of professional significance and value (Brown and Lent, 2012). In line with these findings, Strauss et al. (2012) found that employees with a salient future work self represent the possible positive self in the work situation, which can push the career development of employees. Self-management is the first step in career development and may emerge when employees are eager to take actions designed to fulfill career goals (Locke and Latham, 1990; Turner et al., 2020). When employees detect that their behaviors are conducive to achieving personal goals or match their roles at work, they are more willing to produce self-management (Locke and Latham, 1990).

Second, by making individuals have a more optimistic view of the future and greater confidence in achieving their goals, the future work self can meet the basic needs of individuals, thus enhancing the self-management of individuals. Deci and Ryan (1985) held that employees are more likely to be motivated to participate in an activity when they have a sense of confidence or competence. The employee who has a salient future work self is more optimistic and confident about themselves at work in the future (Strauss et al., 2012). In addition, self-management comes from the willingness of employees to control their behavior without supervision (Breevaart et al., 2014), aimed at obtaining desired behavior (Zeijen et al., 2018). The self-confidence of work and the expectation of positive results can help individuals to motivate goals and actions, control work behaviors, and improve individual self-management (Ireland and Lent, 2018).

We further suggested that the impact of future work self on self-management leads to increased employee workplace wellbeing. Individual wellbeing is relevant to positive affect (Urry et al., 2004), which refers to a pleasant response to the environment. The affect of an individual is predictable in the daily process of self-management. At the core, influencing factors of employee workplace wellbeing cause emotional processes, and the cognitive assessment of emotions promotes mental health experiences. The literature supports the view that career-related effort put into work serves to enhance employee workplace wellbeing (Shimazu et al., 2015). Jung and Takeuchi (2018) revealed that a person who engages in self-management facilitates access to career success resources, such as goal clarity and networks, which lead to increased wellbeing. The clearer the vision of a person is, the more likely they represent themselves in the future regarding aspirations with work and be motived to engage in self-management. The positive aspects of self-management related to need fulfillment and positive effect will extend the vocational focus of psychology on promoting wellbeing in work settings. In line with this, the future work self will be positively associated with self-management and positively associated with employee workplace wellbeing. We thus hypothesized the following:

Hypothesis 2: Future work self will be indirectly and positively related to employee workplace wellbeing through self-management.

### Moderating Effect of Person–Organization Fit

We suggested that the person–organization fit moderates the impacts of future work self on self-management. The term “person–organization fit” refers to the compatibility between the individual and the organization, depending on how well their characteristics match (Kristof-Brown et al., 2005). This concept describes the values, beliefs, and personality characteristics of employees and the degree to which they match the values, beliefs, and norms of the organization. The person–organization fit reflects the similarity of values between them and is an important factor in the work environment that employees count on when they categorize the self and adjust their self-concepts (Kilroy et al., 2017).

High person–organization fit enables individuals to act with the organizational lock-in effect, which is conducive to cultivating the vision of individuals on career development (Sirén et al., 2018). While self-management consists of the control of employees to envision themselves making career development, high person–organization fit expedites the creation of such visions. When the workplace context is conducive to meeting the basic psychological needs of individuals, individuals are more likely to form active behavioral motivations, which in turn facilitate or thwart personal attitudes (Deci et al., 2017). With high person–organization fit, the psychological needs of individuals are more easily met, resulting in more actively engaged work (Rodrigues et al., 2015). Thus, we can view high person–organization fit as allowing the fulfillment of basic needs and development, and low person–organization fit as limiting such fulfillment.

When there is a low level of person–organization fit, employees think that the values of the organization are inconsistent with their values and that they limit their professional development (Hsu et al., 2019). Low person–organization fit establishes a quandary environment, which can lead to negative emotions and hinder actions that help employees achieve their goals (Edwards and Cable, 2009). Low person–organization fit as a social context may thwart individual attitudes. In this circumstance, employees may question the organization and cannot find themselves valuable at work. As a result, employees do not have the incentive to manage themselves because the low level of person–organization fit inhibits the effect of future work self on the self-management of employees. Thus, we proposed the following two hypotheses.

Overall, we proposed that future work self will stimulate the behavioral changes of employees in relation to themselves, increasing their self-management and thus enhancing employee workplace wellbeing. Lin and Chan (2020) hold that wellbeing is always affected by the motivation of an individual as a subjective emotional experience. We suggested that this behavioral impact is more prominent for employees with high person–organization fit because they have more satisfaction regarding their basic needs and development. Employing a self-determination approach, a social environment, such as a workplace that supports meeting basic psychological needs, contributes to autonomous motivation and mental and physical health (Deci and Ryan, 2000). As a result, when the person–organization fit is high, consistent cognitions caused by future work self can create positive cognition, and thus, employee workplace wellbeing emerges. Employees with low person–organization fit may not clearly understand the organization and its relationships (Ashforth et al., 2008), dampening the positive effects of future work self on self-management and employee workplace wellbeing. We proposed the hypothesis as follows:

Hypothesis 3: The person–organization fit will moderate the first stage of the mediated relationship between future work self and employee workplace wellbeing, such that the positive indirect effects through self-management will be stronger when person–organization fit is high.

Combining the above-mentioned hypotheses, Figure 1 shows the proposed moderated mediation model to test future work self and employee workplace wellbeing. The model incorporates self-management as a mediator and the person–organization fit as a moderator.

## Methods

### Sample and Data Collection

The distribution and recovery time of the survey was from April 2019 to May 2019. To minimize common method biases and enhance sample representation, we collected the data from full-time employees from different regions and industries (Podsako et al., 2003). The sample enterprises are mainly in the north of China (i.e., Harbin, Changchun, Shenyang, Fushun, and other cities) in manufacturing, information, education, biological, and pharmaceutical technology. Before the formal survey, we selected some enterprises for a pre-survey. Using the feedback information from the pre-survey, we adjusted unclear survey items so that the respondents could clearly understand the meaning of each item.

To reduce the common method deviation, we divided the survey into two phases. In phase 1, the questionnaire included only questions on the basic information of the respondents and future work self, self-management, and the person–organization fit, which were evaluated by the employees. In phase 2 (i.e., a month later), a questionnaire on employee workplace wellbeing was distributed to those who responded with valid answers during the first stage. We matched and combined the two questionnaires collected from each participant to form a complete questionnaire. We approached 380 full-time employees in phase 1. After phase 2, 250 questionnaires were matched. We excluded invalid questionnaires that were not filled in carefully (i.e., multiple items with the same result), thus obtaining 239 valid questionnaires. The effective questionnaire recovery rate was 62.89%. The sample included 51.90% males and 48.10% females. The average age and average job tenure were 30.25 and 6.92 years, respectively.

### Measurement

To ensure the reliability of the measurement tools, all the measurement tools used in this study are the scales used in the existing literature published in authoritative journals. A 5-point Likert response scale was used, ranging from 1 (i.e., strongly disagree) to 5 (i.e., strongly agree).

#### Future Work Self

Future work self was assessed by using the four-item scale suggested by Guan et al. (2014), which was adapted from the study of Strauss et al. (2012). Employees participating in the survey were asked to envision hoped-for work in the workplace and what they want to do in the future. Keeping this in mind, the items were rated in the survey. A sample item is “This future is very easy for me to imagine.” The Cronbach's alpha is 0.83.

#### Self-Management

We measured self-management using the three-item scale suggested by Renn et al. (2011), which was adapted from the study of Williams et al. (1992). Sample items are “I translate my work goals into action,” “I follow through with achieving my work goals,” and “When it comes to my work goals, action speaks louder than words.” The Cronbach's alpha is 0.72.

#### Person–Organization Fit

We measured person–organization fit using the three-item scale suggested by Cable and Derue (2002). A sample item is “My personal values match my organization's values and culture.” The Cronbach's alpha is 0.77.

#### Employee Workplace Wellbeing

We measured employee workplace wellbeing using the six-item scale suggested by Zheng et al. (2015). A sample item is “I am satisfied with my work responsibilities.” The Cronbach's alpha is 0.84.

#### Control Variables

According to the earlier study, we selected age, gender (0 = female, 1 = male), education, and tenure as control variables because they potentially affect the cognitions and attitudes of the Chinese employees at work (Hui and Tan, 1996; Ng and Feldman, 2012).

## Data Analysis and Results

In this study, Mplus 7.0 and SPSS 26.0 were used for the statistical analysis. By using Mplus 7.0, the confirmatory factor analysis (CFA) was performed to examine construct validity. SPSS 26.0 was used to perform descriptive statistics. Concerning the analyses of mediation and moderated mediation, we used Models 4 and 7 of the Process macro of SPSS 26.0 to test the mediation effect and the moderated mediation effect, respectively (Cohen et al., 2014).

### Confirmatory Factor Analyses

Table 1 provides the results of CFA. Using Mplus 7.0, CFA was performed. The statistics show an acceptable fit of the hypothesized four-factor model comprising future work self, self-management, employee workplace wellbeing, and person–organization fit, which was better to the alternative models (i.e., χ^2^ = 71.46, *df* = 24, CFI = 0.95, TLI = 0.92, RMSEA = 0.01, SRMR = 0.04). The fit of the three-factor model combing self-management and person–organization fit into one factor was as follows: χ^2^ = 120.91, *df* = 24, CFI = 0.90, TLI = 0.84, RMSEA = 0.13, SRMR = 0.08. The fit of the two-factor model combing self-management, person–organization fit, and future work self into one factor was as follows: χ^2^ = 265.66, *df* = 26, CFI = 0.74, TLI = 0.64, RMSEA = 0.20, SRMR = 0.11. The fit of the one-factor model combing self-management, person–organization fit, future work self, and employee workplace wellbeing into one factor was as follows: χ^2^ = 297.18, *df* = 27, CFI = 0.71, TLI = 0.61, RMSEA = 0.21, SRMR = 0.12.

**Table 1:** Results of confirmatory factor analysis.

	**χ^2^**	***df***	**CFI**	**TLI**	**RMSEA**	**SRMR**
Four-factor model: FWS, WB, SM, POF	71.46	24	0.95	0.92	0.01	0.04
There-factor model: FWS, WB, SM + POF	120.91	24	0.90	0.84	0.13	0.08
Two-factor model: FWS+ SM + POF, WB	265.66	26	0.74	0.64	0.20	0.11
One-factor model: FWS + WB + SM + POF	297.18	27	0.71	0.61	0.21	0.12

*n = 239. FWS, future work self; WB, wellbeing; SM, self-management; POF, person–organization fit*.

### Composite Reliability and Average Variance Extracted

The convergent validity and discriminant validities were tested using the measures of composite reliability (CR) and average variance extracted (AVE). According to Bagozzi and Yi (1988), the values of AVE should be above 0.50. The CR values greater than a threshold value of 0.60 are acceptable (Peterson and Kim, 2013). The results show that all the indices have achieved acceptable values (Table 2). Furthermore, according to Fornell and Larcker (1981), if the square root of the AVE from the construct is greater than the correlation shared between the construct and other constructs in the model, the discriminant validity is considered sufficient. Table 2 shows that the square root of the AVE of each construct is greater than the levels of correlations involving that construct, therefore confirming discriminant validity.

**Table 2:** Descriptive statistics, correlations, and square roots of average variance extracted (AVE).

	***Mean***	***SD***	**1**	**2**	**3**	**4**	**5**	**6**	**7**	**8**
1. Gender^a^	0.50	0.50								
2. Education^b^	2.22	0.67	−0.08							
3. Age	30.25	7.43	0.09	−0.06						
4. Tenure	6.92	7.03	0.08	−0.32^**^	0.84^**^					
5. Future work self	3.62	0.90	−0.07	0.12	0.05	0.07	(0.62)			
6. Self-management	3.98	0.58	−0.08	0.03	0.15^*^	0.21^**^	0.47^**^	(0.62)		
7. Person–organization fit	3.41	0.90	−0.04	0.04	0.22^**^	0.25^**^	0.25^**^	0.36^**^	(0.54)	
8. Wellbeing	3.78	0.68	−0.06	0.08	0.15^**^	0.18^**^	0.26^**^	0.44^**^	0.66^**^	(0.54)
CR							0.87	0.91	0.78	0.77

*n = 239; CR, composite reliability*.

a *Gender: 0 = Male; 1 = Female*.

b *Education: 1 = high school or below; 2 = junior college degree; 3 = bachelor's degree, and 4 = master's degree or higher*.

* *p < 0.05*,

** *p < 0.01*.

*The square root of AVEs appear in parentheses along the diagonal*.

### Correlation Analysis of Sample Variables

Table 2 shows the descriptive statistics, correlations, and scale reliabilities. The results show that the future work self was positively related to self-management (*r* = 0.47, *p* < 0.01) and employee workplace wellbeing (*r* = 0.26, *p* < 0.01). Self-management was positively related to employee workplace wellbeing (*r* = 0.44, *p* < 0.01).

### Hypothesis Testing

Table 3 shows the regression results. The results revealed that the future work self was positively related to employee workplace wellbeing (Model 5, β = 0.17, *p* < 0.01). Thus, Hypothesis 1 was supported. In addition, we used the bootstrapping method for assessing mediation (Cheung and Lau, 2008). For Hypothesis 2, the 95% bias-corrected bootstrap CIs were determined for the assumed indirect effect from 5,000 bootstrap samples. As shown in Table 4, the correlation between future work self and employee workplace wellbeing through self-management was as follows: indirect effect = 0.127, SE = 0.029, 95% CI (0.074, 0.187), which is significant. Thus, the Hypothesis 2 was supported.

**Table 3:** Summary of the results of the multiple regression analysis.

	**Self-Management**		**Wellbeing**			
	**Model 1**	**Model 2**	**Model 3**	**Model 4**	**Model 5**	**Model 6**
**Control variables**						
Gender^a^	−0.10	−0.07	−0.07	−0.09	−0.06	−0.04
Education^b^	0.13^*^	0.05	0.03	0.17^*^	0.12^*^	0.10
Age	−0.02	−0.01	−0.01	−0.01	−0.01	−0.01
Tenure	0.04^**^	0.03^*^	0.02^*^	0.03^*^	0.03^*^	0.01
**Independent variable**						
Future work self		0.29^**^	0.26^**^		0.17^**^	0.04
**Moderator**						
Person–organization fit			0.15^**^			
**Interaction**						
Future work self × person–organization fit			0.08^*^			
**Mediator**						
Self-management						0.45^**^
**R**^**2**^	0.08	0.26	0.32	0.06	0.11	0.21
**adjusted-R**^**2**^	0.06	0.25	0.30	0.04	0.09	0.19
**ΔF-statistic**	4.72^*^	59.59^**^	14.49^**^	3.66	12.60^**^	31.51^**^
**ΔR**^**2**^		0.19^**^	0.02^*^		0.05^**^	0.11^**^

*n = 239*.

a *Gender: 0 = Male; 1 = Female*.

b *Education: 1 = high school or below; 2 = junior college degree; 3 = bachelor's degree and 4 = master's degree or higher*.

* *p < 0.05*,

** *p < 0.01*.

**Table 4:** Indirect effects of future work self on wellbeing.

**Path**	**Effect**	**SE**	**95% CI**
Direct: Future work self → wellbeing	0.042	0.050	[−0.057, 0.141]
Indirect: Future work self → self-management → wellbeing	0.127^**^	0.029	[0.074, 0.187]
Total: Direct + indirect	0.169^**^	0.048	[0.075, 0.262]

*n = 239*.

* *p < 0.05*,

** *p < 0.01 (two-tailed)*.

For Hypothesis 3, this study tested whether the person–organization fit moderate the relationship between future work self and employee workplace wellbeing through self-management. We calculated the conditional mediating effect of self-management at different levels of person–organization fit. In this study, we used Model 7 of the Process macro of SPSS 26.0. As shown in Table 5, when the person–organization fit was higher (at +1 SD of mean), the indirect effect of future work self on employee workplace wellbeing through self-management was significant [*b* = 0.153, boot SE = 0.028, 95% bias-corrected CI = (0.074, 0.184)]. When the person–organization fit was lower (at −1 SD of mean), the indirect effect of future work self on employee workplace wellbeing through self-management was significant [*b* = 0.084, boot SE = 0.028, 95% bias-corrected CI = (0.035, 0.145)]. The index of moderated mediation was significant [*b* = 0.041, boot SE = 0.022, 95% bias-corrected CI = (0.002, 0.087)]. Hence, Hypothesis 3 was supported.

**Table 5:** Conditional indirect effects.

**Variables**	**Wellbein**g		
	**Estimate**	**SE**	**95% CI**
Low person–organization fit	0.084*	0.028	[0.035, 0.145]
High person–organization fit	0.153^**^	0.028	[0.074, 0.184]
Index of moderated mediation	0.041*	0.022	[0.002, 0.087]

*n = 239*.

** *p < 0.01 (two-tailed)*.

## Discussion

The prevalence of future work self among employees creates challenges in managing organizations efficiently. We explored the positive effect of future work self on employee workplace wellbeing, the mediating effect of self-management, and the moderating effect of person–organization fit in this relationship. Based on the self-determination theory, using a time-lagged analysis on 239 employees from Chinese enterprises, this study clarifies how, why, and when future work self may lead to employee workplace wellbeing in the workplace.

### Theoretical Implications

Our findings have several notable theoretical implications. First, we extended the literature of employee workplace wellbeing by identifying future work self as a potential antecedent of employee workplace wellbeing. Prior study has explored many individual motivational resources in predicting employee workplace wellbeing (Nielsen et al., 2017; Boncquet et al., 2020), such as psychological capital (Luthans and Youssef, 2004), core self-evaluations (Bipp et al., 2019), and achievement goals (Heidemeier and Wiese, 2014). However, whether future work self, as a new concept that can be viewed as an eminent motivational resource (Strauss et al., 2012), benefits work-related outcomes, especially employee workplace wellbeing, still needs to be explored. Earlier studies have shown that having a salient future work self is conducive to positive outcomes (Guan et al., 2014, 2017; Lin et al., 2016; Yang et al., 2019). This study extends this line of research by recognizing future work self as an important antecedent for employee workplace wellbeing. This extension is meaningful because it suggests that future work self, i.e., a self-concept that relates to the hopes in individuals for a future work role (Strauss et al., 2012), can enable them to meet their basic psychological needs in the process of pursuing their ideal self and thus affect their attitudes and behaviors. Therefore, the evidence that this study offers sheds additional light on the role of motivational antecedents in influencing employee workplace wellbeing and supports the positive impact of future work self.

Second, the mediating effect of self-management between future work self and employee workplace wellbeing was further clarified and confirmed. The linkage from future work self to employee workplace wellbeing and the underlying mediating effect of self-management have not been established. The self-determination perspective widens the scope of explanation for the effects of future work self by realizing the gap between the present and the future, focusing on the positive outcomes of employee workplace wellbeing. For employees with a high level of future work self, their long-term work goals are more closely aligned with their immediate work goals, and this may enhance their dedication (Lin et al., 2016). Moreover, the identification of behavior–goal discrepancies can lead to an increase in goal-directed behavior (Kernan and Lord, 1990), which in turn enhances employee workplace wellbeing. Studies have shown that work investment by individuals is positively related to employee workplace wellbeing (Shimazu et al., 2015). Hence, this study contributes to the literature of future work self by identifying the process that can reveal how future work self positively influences employee workplace wellbeing through self-management.

Third, by applying a moderated mediation framework, we revealed the moderating role of the person–organization fit, supporting the applicability of a self-determination perspective in explaining the link between future work self and employee workplace wellbeing through self-management. This conclusion is consistent with the finding in the literature that the person–organization fit has a positive interaction effect on satisfaction (Liu et al., 2015). Employees who work in an environment that provides person–organization fit have highly positive job attitudes (Vianen and Annelies, 2018), which is more conducive to their career development. Thus, we can view high person–organization fit as part of a positive external environment. We further proposed that high levels of person–organization fit will strengthen the positive impact of employee self-management and employee workplace wellbeing. Our findings provide insights into how person–organization fit, as a contextual factor to facilitate self-determination, can boost the increased effort of employees into current work outcomes (i.e., self-management).

### Practical Implications

Simultaneously, this study offers specific practical contributions. First, our empirical results demonstrated that future work self is associated with employee workplace wellbeing. Therefore, enterprises should promote and train salient future work self among employees, which might foster employee workplace wellbeing. For example, enterprises need to pay attention to the characteristics and professional values of employees. When mentoring employees, enterprise managers can help employees to clarify their future work self by specifying their future work expectations and visions, which in turn may motivate individuals to actively engage in career-discovery behaviors and work and thus increase their employee workplace wellbeing.

Second, given the findings of this study, self-management can serve as a mediator in the relationship between future work self and employee workplace wellbeing. We suggest that employees should aim to improve their self-clarity by paying attention to their possible selves in their future careers to enhance self-management. Enterprises should also give careful consideration to creating environments that foster the improvement of self-management. For example, they can implement training or coaching interventions focusing on promoting self-management of individuals.

Third, considering the moderating effect of person–organization fit, we suggest that organizational values need to be integrated into the values of employee training. That is, when fostering the values of employees, managers should attach importance to the input of corporate culture, realize appropriate value-matching between people and the organization, and help employees adapt more effectively to the organization.

## Limitations and Future Research

There are some limitations in this study. First, although the data were collected from multiple sources and a time-lagged design was used, the current data may have reverse causality. Employees with higher levels of employee workplace wellbeing may be more likely to have a conception of their future work self. To improve the causal direction of the model in this research, a longitudinal way is needed in future research. Second, the data sources of this study are not diverse. This research collected data only from the Chinese enterprises, and hence, all participants had the same cultural background. Future research might consider replicating the findings of this study in different cultural contexts. Third, this study utilizeed a limited number of variables. Future studies ought to contemplate including more contextual factors to explore their influence on the behavioral outcomes of future work self. In particular, a study that extends the fundamental theory of self-determination argues that contextual conditions that promote autonomous motivation can facilitate people to motivate themselves autonomously and, in turn, work well and feel good (Deci et al., 2017). Finally, as one of the most important contextual conditions for employees, leader behavior will inevitably influence the perceptions and behaviors of employees. Thus, future research should consider various leadership styles to explain the boundary conditions of future work self and employee workplace wellbeing.

## Conclusion

Employee workplace wellbeing is the overall quality of experience of an employee that can improve organizational effectiveness. To maintain competitive advantage of the enterprises, organizations increasingly adopt multiple methods to enhance employee wellbeing, such as an opportunity for skill use, a variety at work, environmental clarity, availability of money, and physical security (Guest, 2017). This study extends the literature on employee workplace wellbeing by identifying the role of future work self in improving employee workplace wellbeing. To clarify this mechanism, we employed self-management as the mediator. Our findings revealed that the salient future work self can improve self-management, thereby increasing employee workplace wellbeing, especially when employees have high levels of person–organization fit.

## Data Availability Statement

The data that support the findings of this study are available from the corresponding author upon reasonable request.

## Ethics Statement

Ethical review and approval was not required for the study on human participants in accordance with the local legislation and institutional requirements.

## Author Contributions

Z-QL and YY conceived the idea of the study. Z-QL and XZ collected the data and wrote the manuscript. All authors discussed the results and revised the manuscript.

## Conflict of Interest

The authors declare that the research was conducted in the absence of any commercial or financial relationships that could be construed as a potential conflict of interest.
